# Platelet-rich plasma for the treatment of lichen sclerosus

**DOI:** 10.20517/2347-9264.2021.86

**Published:** 2021-12-05

**Authors:** Beija K. Villalpando, Saranya P. Wyles, Lauren A. Schaefer, Katherine J. Bodiford, Alison J. Bruce

**Affiliations:** 1 Mayo Clinic Alix School of Medicine, Rochester, MN 55905, USA.; 2 Department of Dermatology, Mayo Clinic, Rochester, MN 55905 USA.; 3 Department of Dermatology, Mayo Clinic, Jacksonville, FL 32224, USA.

**Keywords:** Platelet rich plasma, lichen sclerosus, vulvar lichen sclerosus, penile lichen sclerosus

## Abstract

**Aim:**

Evaluate the clinical effectiveness of platelet-rich plasma as a treatment for lichen sclerosus.

**Methods:**

A systematic review was performed. The electronic databases PubMed, Ovid MEDLINE^®^, Web of Science, Cochrane, clinicaltrials.gov were used to identify case studies, case series, prospective uncontrolled, and randomized controlled studies published between 1946 and April 21, 2021. Six prospective uncontrolled studies, one randomized double-blind prospective study, and one case report were included.

**Results:**

Platelet-rich plasma treatment was subjectively reported to improve quality of life, but objective measures demonstrating treatment efficacy were not observed. In addition, platelet-rich plasma preparation and administration between studies lacked standardization.

**Conclusion:**

Platelet-rich plasma may be used for symptomatic adjuvant treatment of lichen sclerosus, though additional double-blind controlled studies with standardized platelet-rich plasma protocols are needed to better characterize the efficacy of platelet-rich plasma.

## INTRODUCTION

**Figure 1.**
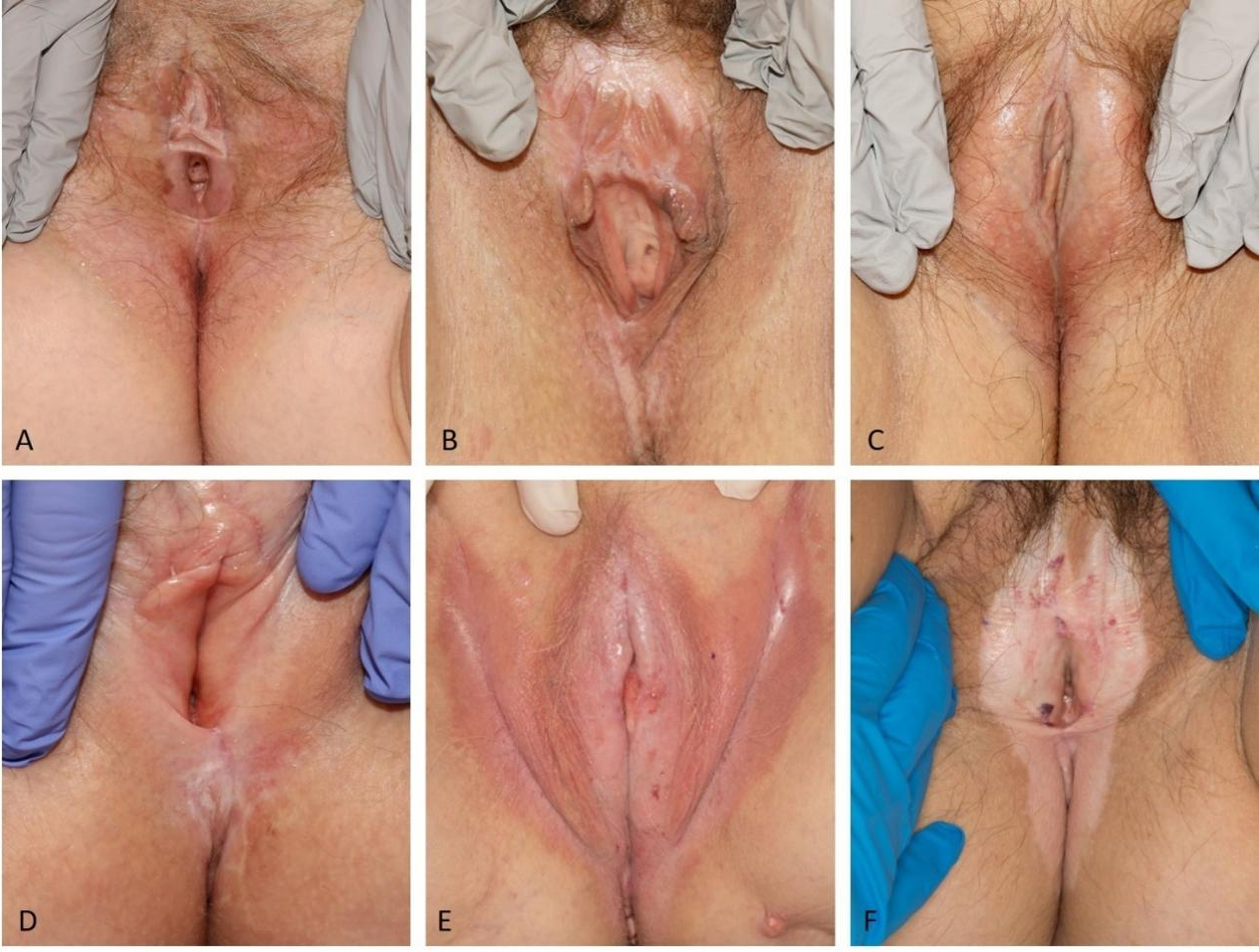
Patients with vulvar LS. Figure 1A shows a white atrophic patch involving the vulva and perianal skin in a figure-of-eight distribution with epidermal atrophy, agglutination of the bilateral labia minora, and superficial erosions present at the introitus. Figure 1B shows erythematous to hypopigmented vulvar patches with agglutination. Figure 1C shows atrophic white and focal erythematous patches without erosion or purpura. Figure 1D shows a white atrophic patch with minimal erythema in the vulva, agglutination with loss of the labia minora, and scarring of the clitoral hood. Figure 1E shows confluent erythematous patches involving the labia and the inguinal folds with whitish atrophic areas, minor fissures and cracks. Figure 1F shows hypopigmentation in a figure-of-eight distribution involving the vulva and perianal area with scattered areas of hemorrhage. (By permission of Mayo Foundation for Medical Education and Research. All rights reserved.)

**Figure 2.**
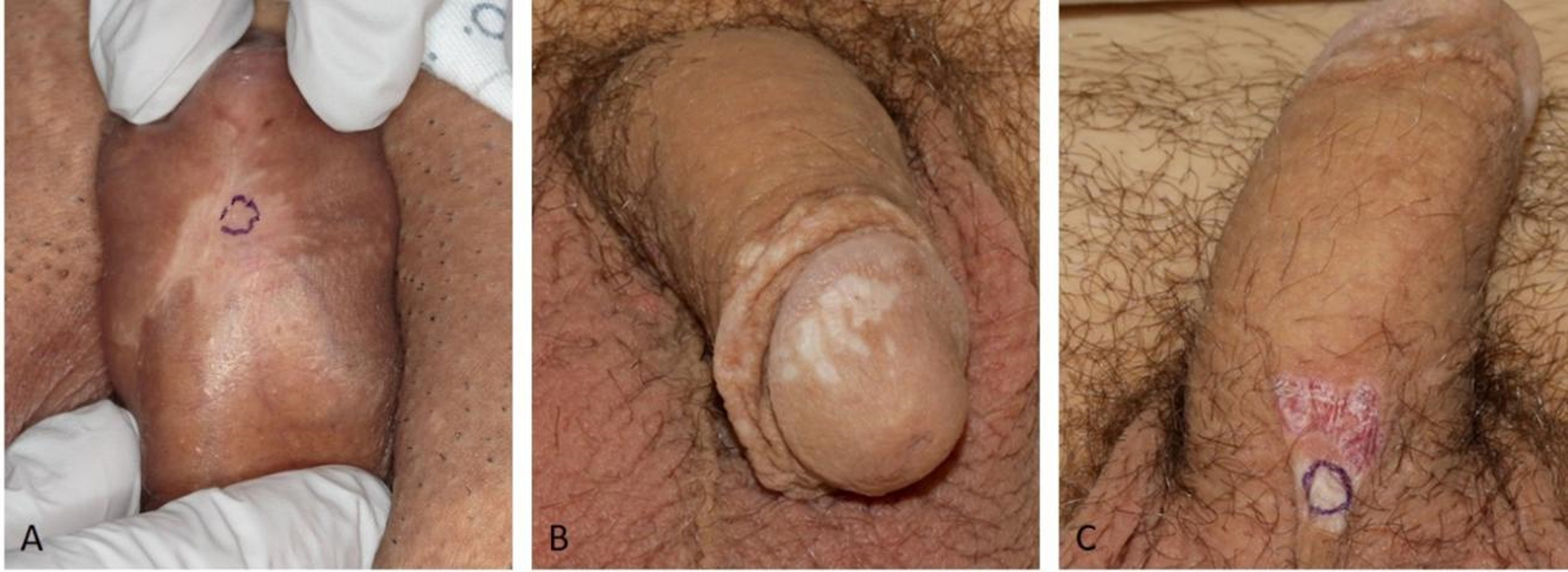
Patients with penile LS. Figure 2A shows a hypopigmented, atrophic, slightly geometrically shaped patch involving the ventral penis. There is a purple biopsy mark site present. Figure 2B shows numerous scattered white atrophic macules coalescing to patches. Figure 2C shows a white atrophic patch with epidermal wrinkling and hemorrhage on the ventral base of the penis. There is a purple biopsy mark site present. (By permission of Mayo Foundation for Medical Education and Research. All rights reserved.)

Lichen sclerosus (LS) is a chronic inflammatory dermatosis characterized by ivory-white patches with surface wrinkling due to epidermal atrophy, which may display areas of hemorrhage or scar-like changes. Lichen sclerosus commonly affects the anogenital region in males and females [Figure 1–2], although extragenital presentations also occur^[1]^. It is estimated to occur in 1 in 30 women and 1 in 900 pre-pubertal girls^[2]^. The etiology of LS has not yet been elucidated, though is likely multifactorial, and may include autoimmune, genetic, and hormonal causes including estrogen deficiency^[3,4]^. Chronic trauma or irritation may be contributory^[5]^.

There is currently no cure for LS. Accepted first-line treatment for LS is daily application of potent topical corticosteroids such as clobetasol propionate 0.05% ointment^[6,7]^. Long-term topical steroid usage is potentially limited by compliance, side effects including further skin atrophy, steroid tachyphylaxis and steroid-induced dermatitis^[8]^. For topical steroid treatment-resistant LS, alternate topical, intralesional and systemic therapies are utilized, i.e., topical calcineurin inhibitors, topical and systemic retinoids, intralesional corticosteroid injection, systemic immunosuppressants, and phototherapy. However, these second- and third-line therapies can be associated with poor initial response, resistance over time, and/or adverse drug effects. Further investigation regarding the efficacy of these treatments is warranted^[9]^. Currently, autologous platelet-rich plasma (PRP) is being explored as a treatment for LS.

**Figure 3.**
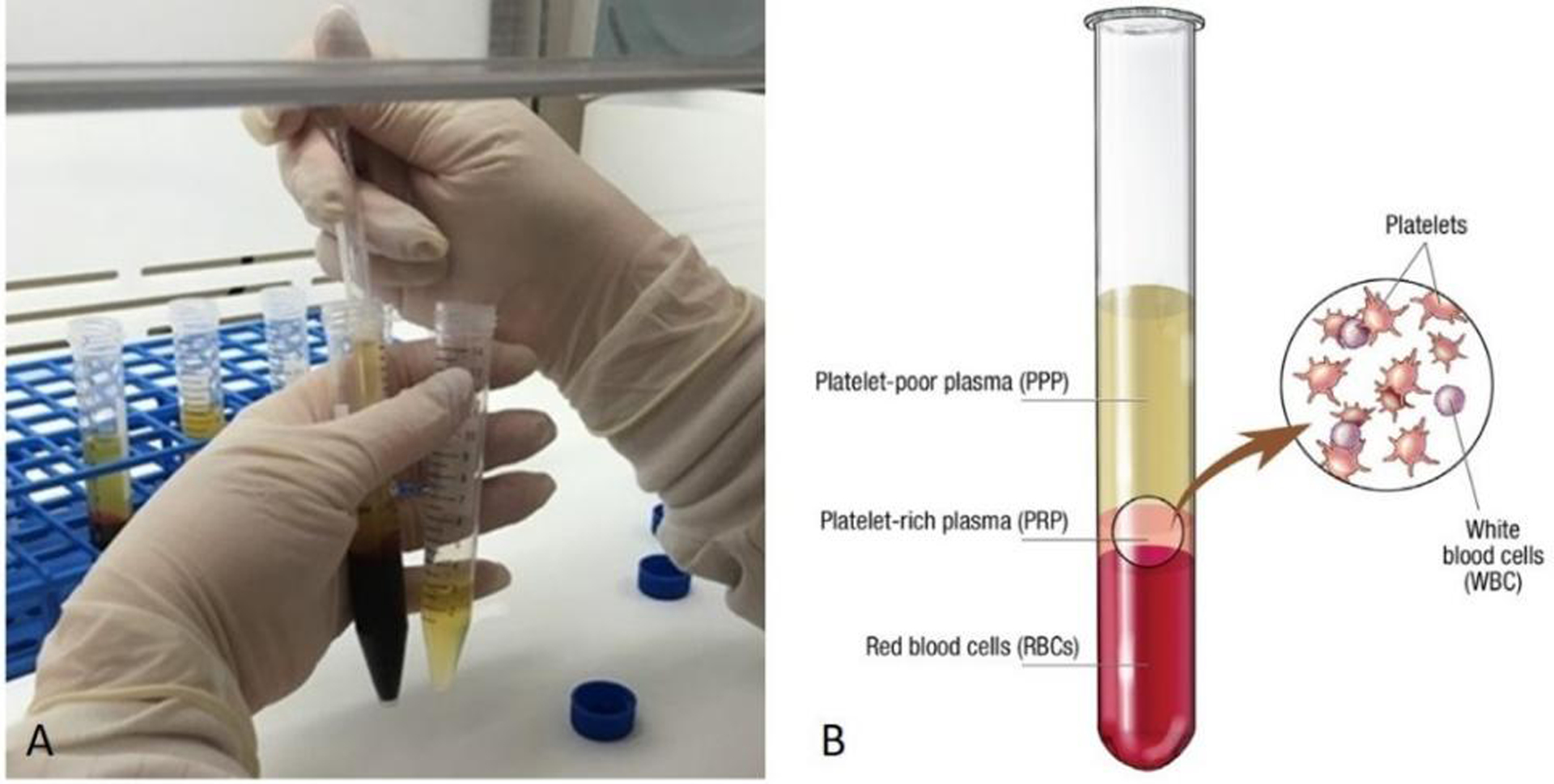
PRP processing. Figure 3A shows removal of the upper portion of plasma from each conical tube after centrifugation. Figure 3B shows separation of red blood cells, platelet-rich plasma, and platelet poor plasma following centrifugation. PRP: Platelet Rich Plasma. (By permission of Mayo Foundation for Medical Education and Research. All rights reserved.)

Autologous PRP is obtained from the patient’s own blood sample, which is drawn at the time of treatment. The blood draw occurs with the inclusion of an anticoagulant, such as citrate dextrose A, to prevent blood clotting during preparation. The patient’s blood is then centrifuged, and the first spin is performed at constant acceleration to separate red and white blood cells from the remaining whole blood volume. The supernatant plasma containing platelets is then transferred into a sterile tube and again centrifuged at a higher speed, allowing for a concentrated platelet level to form in the lower tube[Figure 3]. The upper third of platelet-poor plasma is then removed, leaving the platelet-rich plasma to be administered^[10]^. Platelet-rich plasma contains a high level of growth factors, which are important for regenerative and therapeutic treatment. More specifically, PDGF and TGF-β stimulate fibroblast proliferation to increase collagen production^[11]^. Platelet-rich plasma increases the expression of matrix metalloproteinases (MMPs) which modulate remodeling^[11]^. Transforming GF alpha (TGF-α) and EGF have been shown to modulate keratinocyte propagation and migration to thicken the epidermis^[11]^.

While PRP was initially used by hematologists in the 1970s as a transfusion treatment for thrombocytopenia, the intrinsic regenerative properties of PRP has broadened its therapeutic use^[12]^. In the 1980s, PRP first emerged from a strictly thrombocytopenic treatment to a peri-operative treatment for maxillofacial surgery^[12]^. However, PRP has been most extensively studied as a therapy for musculoskeletal injuries, including chronic tendinopathies, acute ligamentous injuries, muscle injuries, osteoarthritis, and bone healing for various types of fractures^[12,13].^ Since PRP’s therapeutic inception, it has been utilized in many medical fields, such as dermatology, cardiovascular surgery, orthopedic surgery, pain management, and plastic surgery ^[13]^. Specific to dermatological practice, PRP is being explored as a regenerative therapy for a variety of conditions including wound healing, scar revision, alopecia, and LS^[12]^. The aim of this review is to identify, critically assess and synthesize available scientific evidence on the safety and clinical efficacy of PRP for the treatment of LS. To fulfill this aim, we have conducted a review of the scientific literature.

## METHODS

An experienced information specialist developed and conducted an extensive search of Ovid MEDLINE^®^ 1946 to Present and Epub Ahead of Print, In-Process & Other Non-Indexed Citations and Ovid MEDLINE^®^ Daily, Ovid MEDLINE^®^ Daily Update, EBM Reviews - Cochrane Central Register of Controlled Trials March 2021, EBM Reviews - Cochrane Database of Systematic Reviews 2005 to April 21, 2021, Embase 1974 to 2021 April 21, PubMed 1946 to 2021 April 21, Web of Science 1975 to 2021 April 21, and ClinicalTrials.gov through 2021 April 21 to identify case studies, case series, prospective uncontrolled, and randomized controlled studies published between 1946 and April 21, 2021. The search strategy combined the terms “Lichen Sclerosus et Atrophicus” OR “lichen sclerosus” OR “lichen scleroses” AND “Platelet-Rich Plasma” OR “platelet rich plasma” OR “thrombocyte-rich plasma” OR “thrombocyte rich plasma”. Works were limited to English language and excluded animal studies, guidelines, protocols, and review articles. Potentially eligible titles were identified using controlled vocabulary in tandem with key words. The search strategy was peer-reviewed prior to execution.

## RESULTS

**Figure 4.**
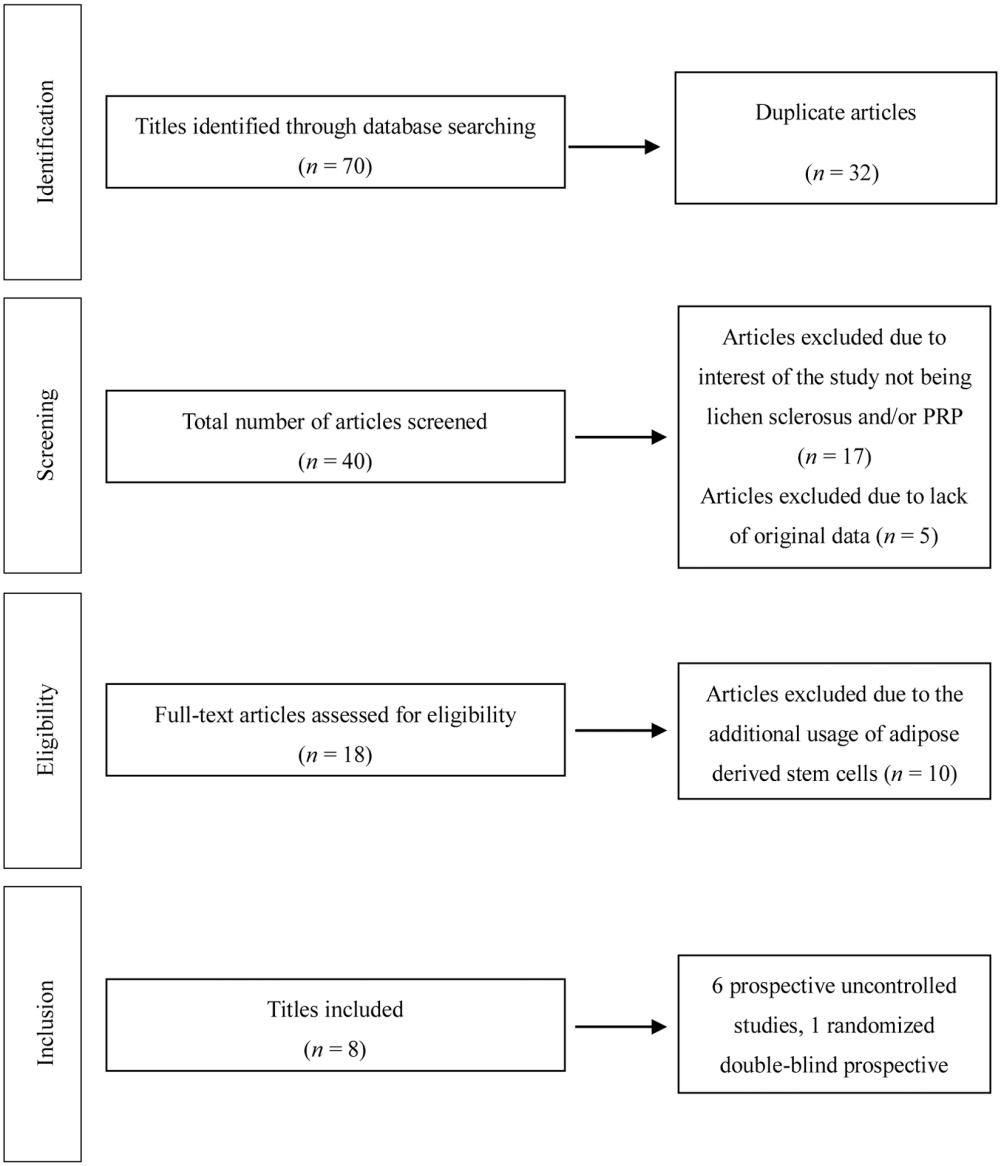
Flow diagram summarizing literature search, screening and review. PRP: Platelet-rich plasma.

The results of the literature search and study selection process are shown in Figure 4. The main characteristics of selected studies are summarized in Table 1. All of them were published in English between 2016 and 2020. Study size was highly variable, ranging from 1 to 94 participant(s). Eight total studies included 6 prospective uncontrolled studies, 1 randomized double-blind prospective study, and 1 case report. Level of evidence ranged from level III to level V. Participants ranged from age 21 to 88 years old. Five studies evaluated only female participants; one study evaluated only male participants; and 3 studies included both female and male participants. Types of LS evaluated include vulvovaginal, vulvar, and penile. Lichen sclerosus diagnosis was established with histopathology (H&E) for 6 studies; histopathology (H&E) combined with colposcopy for 1 study; and not reported for the remaining studies. Four studies evaluated participants who failed prior treatment including topical steroids, emollients, and/or circumcision.

The source of PRP varied between studies and is summarized in Table 2. PRP spin approach, spin duration, and activator were not reported for the majority of studies; only 1 study reported a double-spin approach, 6- and 12-minute spin durations, with calcium chloride as a PRP activator. Four studies reported local anesthesia use prior to PRP application, while the remaining studies did not report if anesthesia was utilized. Seven of the 8 studies reported the PRP amount applied per treatment, which ranged from 2–10 ml. One study doubled the volume in the second dose received, while 6 studies maintained the same PRP dose between treatments. One study did not report PRP amount used. Platelet-rich plasma delivery method varied between studies but included subdermal and intradermal injections into the affected areas. Of the seven studies that reported number of treatments, participants received between 2 to 10 total treatments. Six studies reported standard treatment schedules among participants with an average of 2.98 treatments per participant. Follow-up time ranged from 2 to 23.2 months with an average follow-up time of 9.9 months; three studies did not provide follow-up duration.

Six of the 8 studies used patient questionnaires to assess the effect of PRP on LS symptom relief and/or quality of life. Three studies found that PRP treatment led to full or partial LS symptom relief. Using validated questionnaires, 3 studies reported PRP improved patient quality of life. Posey *et al*.^[19]^ reported the utilization of a patient questionnaire but none of the observed outcomes were clinically significant. Following PRP treatment, Behnia-Willison *et al*., used colposcopy to evaluate vulvovaginal lesions and reported 28% of LS lesions completely resolved. Two studies compared pre- and post-treatment histology.

## DISCUSSION

Therapies rooted in regenerative medicine, such as PRP, seek to repair damaged tissues and restore normal function via stimulating the body’s own regenerative capacity. Platelet-rich plasma consists of various fundamental proteins, which contribute to wound healing including platelet derived growth factor (PDGF), transforming growth factor-β (TGF-β), vascular endothelial growth factor (VEGF), epidermal growth factor (EGF), and adhesive proteins -fibrin, fibronectin, and vitronectin^[22]^. These factors promote angiogenesis, mitogenesis, macrophage activation, vasculogenesis, proliferation, differentiation, and regulation of inflammatory process^[23]^.

Previous studies reported that PRP administration improved quality of life, as well as clinical and histological parameters of LS^[23]^. However, the quality of the evidence is weak. Confidence in the literature is limited due to lack of control and comparison groups, placebo groups, and random assignments. Goldstein *et al*., was the only double-blinded controlled study to assess the efficacy of LS, and this study found no benefit of PRP compared to placebo. In addition, the literature is further limited by the lack of standardization of PRP preparation, quantity, concentration, treatment frequency, and delivery method. Continuing, the literature illustrated a wide variety of outcome measurements with no universally accepted intervention that objectively illustrates PRP benefit in the treatment of LS.

Indeed, LS is a debilitating disease that left untreated causes scarring, discomfort, dyspareunia, decreased quality of life, and potentially increases the risk of cancer development. The main goals for the treatment and management of LS are to provide symptomatic relief, minimize urinary and/or sexual dysfunction and prevent malignant transformation^[24]^. It has not been proven whether early steroid treatment of LS mitigates the risk of malignant transformation, although a recent retrospective review of 301 male patients treated with topical steroids found no progression to penile SCC^[25]^. In addition, a longitudinal prospective cohort study found that long-term topical steroid use decreased scarring and the incidence of vulvar squamous cell carcinoma in women with vulvar LS^[8]^.

Future directions for LS management should focus on expanding treatment options to improve quality of life for affected patients and help prevent malignant transformation. Platelet-rich plasma may prove to be a valuable treatment intervention. However, the variability in preparation and administration needs to be addressed in order to allow comparative assessment. In addition to PRP, regenerative therapies for LS also include adipose-derived stem cells (ADSCs), which are postulated to exert their effect through paracrine release of growth factors^[26]^. Limited studies have evaluated the efficacy of fat grafting in the treatment of severe vulvar LS with varying improvement in mucocutaneous elasticity and resolution of symptoms i.e., pruritus and dyspareunia^[27]^.

The rise in such regenerative therapies warrants further studies to standardize both cellular and acellular clinical adjuvants for wound healing in inflammatory skin conditions, such as LS. Platelet-rich plasma may have a role as a supporting treatment in selected cases of patients with severe LS who do not respond to first line therapy, in severe cases where the anatomical impairment impedes regular sexual function and patient quality of life or where other therapies are poorly tolerated or contraindicated.

## CONCLUSION

Platelet-rich plasma is an emerging regenerative medicine therapy currently being explored as treatment for chronic inflammatory dermatoses including LS. Previous studies, primarily prospective uncontrolled design, demonstrated patient quality of life improvement for LS patients following PRP administration. However, objective and measurable post-treatment improvement from double-blind controlled studies is lacking. In addition, PRP preparation and application is highly variable, which makes treatment efficacy difficult to assess comparatively between studies. While PRP may have a therapeutic role in treatment-resistant LS, high level evidence is needed with standardized PRP processing and application to routinely recommend PRP as an adjuvant treatment for LS.

## Figures and Tables

**Table 1.** Study demographics.

Authors	Type of Study	Level of Evidence	*n* (Females: Males)	Age (year)	Type of LS	LS Diagnosis	Prior Treatment	Outcomes	Follow-up (months)
Behnia-Willison *et al*.^[14]^ (2016)	PU	IV	28 (F)	22–88	Vulvovaginal	Colposcopy ± histopathology	Topical steroids	8/28 (28.5%) LS skin lesions resolved 15/28 (53.6%) LS full symptoms^#^ relief 13/28 (46.4%) LS partial symptoms^#^ relief	12
Casabona *et al*.^[15]^ (2017)	PU	IV	45 (M)	42.96 ± 11.32	Penile	NR	Topical steroids ± circumcision	42/45 (93.3%) resolved phimosis and 44/45 (97.7%) resolved meatus stenosis 45/45 (100%) patients had no atrophy, splitting and inflammation Patients reported improved IGA quality of life (P <0.001) and DLQI (P <0.001)	17.6 ± 5.6
Goldstein *et al*.^[16]^ (2017)	PU	IV	12 (F)	NR	Vulvar	Histopathology	NR	7/12 (58.3%) decreased inflammation in post-treatment skin biopsy 5/12 (41.7%) no change or minimal increase in post-treatment skin biopsy	NR
Franic *et al*.^[17]^ (2018)	CR	V	1 (F)	38	Vulvar	Histopathology	Topical steroids and emollients	Patient reported improved ICIQ-VS for quality of life and sexual matters Post-treatment FSFI improved from 3.6 to 32.6 (range 2 to 36)	2
Goldstein *et al*.^[18]^ (2019)	RDBP	III	29 (F) 10 placebo, 19 PRP	52.6	Vulvar	Histopathology	NR	None of the observed outcomes were statistically significant	NR
Posey *et al*.^[19]^ (2019)	PU	IV	38 (F)	NR	Vulvar	Histopathology	NR	None of the observed outcomes were statistically significant	NR
Tedesco *et al*.^[20]^ (2019)	PU	IV	31 (18F:13M)	NR	Vulvar and penile	Histopathology	Topical steroids	19/31 (62%) reported symptom^#^ relief 11/31 (35%) reported stable symptoms^#^ 1/31 (3%) reported symptom^#^ worsening 76.4% of females reported improvement compared to 47.5% of males (*P* < 0.05)	12
Tedesco *et al*.^[21]^ (2020)	PU	IV	94 (51F:43M)	21–83	Vulvar and penile	Histopathology	NR	Patients reported improvement in itch (58.8%:27.7% F:M), burning (35.3%:21% F:M), pain (25.5%:0% F:M), and dyspareunia (0%:23.3% F:M) Patients reported improved DLQI (*P* < 0.0001)	6

**Table 2.** Platelet-rich plasma preparation and application.

Authors	PRP spin approach	Company	PRP spin duration	PRP activator	Anesthesia	PRP amount applied	PRP application method	PRP application frequency
Behnia-Willison *et al*.^[14]^ (2016)	NR	Regens Lab, New York, N.Y.	NR	NR	Local anesthesia	NR	Affected areas of the external genitalia, including the labia majora, labia minora, clitoris, and clitoral hood was injected using a 27-gauge needle in a fanning motion.	Patients received 3 treatments every 4 to 6 weeks and again at 12 months.
Casabona *et al*.^[15]^ (2017)	Double	NR	6 min at 1000 rpm then 12 min at 3000 rpm	CaCl_2_	Topical and local anesthesia	2 mL per treatment	PRP was applied to ulcerated areas with micro-wheals technique, while subcutaneous and submucosal micro-tunneling technique was performed in sclerotic/fibrotic areas.	Patients received a range of 2–10 treatments (unspecified interval).
Goldstein *et al*.^[16]^ (2017)	NR	Magellan^®^ Autologous Platelet Separator System, Hopkinton, MA	NR	NR	NR	5 mL per treatment	Autologous PRP was applied subdermally and intradermally.	Patients received 2 treatments separated by 6 weeks.
Franic *et al*.^[17]^ (2018)	NR	Cellular Matrix RegenKit^®^ Regen Lab SA Lausanne, Switzerland	NR	NR	Local anesthesia	4 mL with first procedure, 8 mL with second procedure	Autologous PRP was injected subdermally in affected regions.	Patients received 2 treatments separated by 2 months.
Goldstein *et al*.^[18]^ (2019)	NR	Magellan^®^ Autologous Platelet Separator System, Hopkinton, MA	NR	NR	NR	5 mL per treatment	PRP was injected sub-dermally and intra-dermally to affected areas.	Patients received 2 treatments separated by 6 weeks.
Posey *et al*.^[19]^ (2019)	NR	NR	NR	NR	NR	10 mL per treatment	PRP was injected into affected areas.	NR
Tedesco *et al*.^[20]^ (2019)	NR	SELPHYL^®^, Cascade Medical Enterprises, Plymouth, UK	NR	NR	NR	4 mL per treatment	In females, PRP was injected into posterior fourchette (2 wheals), hood (2 wheals) and into the right and left labia (2 wheals). In males, 4 mL was injected into the four cardinal points of the area affected with microwheals technique.	Patients received 1 treatment every 15 days for 3 total treatments.
Tedesco *et al*.^[21]^ (2020)	NR	C.Punt-Biomed System and SELPHYL^®^, Cascade Medical Enterprises, Plymouth, UK.	NR	NR	Topical anesthesia	4 mL per treatment	PRP was injected into the posterior fourchette (2 wheals), into the hood (2 wheals) and into labia minora (2 wheals).	Patients received 1 treatment every 15 days for 3 total treatments.

^ PU: Prospective uncontrolled; RP: Randomized prospective; CS: Case series; CR: Case report; RDBP: Randomized double-blind prospective; NR: Not recorded.

# Symptoms: itch (requiring steroid treatment), soreness, discomfort, burning and/or dyspareunia.

FSFI: Female Sexual Function Index evaluated desire, arousal, lubrication, orgasm, satisfaction, and pain. DLQI: Dermatology Life Quality Index evaluated itch, pain, and feelings of embarrassment and self-consciousness, problems with therapy, and interference from a skin disease in daily activities, relationships, and sex life. IGA: Investigator’s Global Assessment evaluated extent of disease involvement based on erythema, infiltration, lichenification, and excoriation. ICIQ-VS: Incontinence Questionnaire -Vaginal Symptoms.

## References

[1] Röcken M, Ghoreschi K. Morphea and Lichen Sclerosus. In: Bolognia JL, Schaffer JV, Cerroni L, editors. Dermatology. China: Elsevier Limited; 2018. p 707–21.

[2] Marfatia Y, Surani A, Baxi R. Genital lichen sclerosus et atrophicus in females: An update. Indian J Sex Transm Dis AIDS 2019;40:6–12. 31143853 10.4103/ijstd.IJSTD_23_19 PMC6532494

[3] Sherman V, McPherson T, Baldo M, Salim A, Gao XH, Wojnarowska F. The high rate of familial lichen sclerosus suggests a genetic contribution: an observational cohort study. J Eur Acad Dermatol Venereol 2010;24:1031–4. 20202060 10.1111/j.1468-3083.2010.03572.x

[4] Nair PA. Dermatosis associated with menopause. J Midlife Health 2014;5:168–75. 25540566 10.4103/0976-7800.145152 PMC4264279

[5] Hofer MD, Meeks JJ, Mehdiratta N, Granieri MA, Cashy J, Gonzalez CM. Lichen sclerosus in men is associated with elevated body mass index, diabetes mellitus, coronary artery disease and smoking. World J Urol 2014;32:105–8. 23633127 10.1007/s00345-013-1090-7

[6] Neill SM, Lewis FM, Tatnall FM, Cox NH; British Association of Dermatologists. British Association of Dermatologists’ guidelines for the management of lichen sclerosus 2010. Br J Dermatol 2010;163:672–82. 20854400 10.1111/j.1365-2133.2010.09997.x

[7] Dalziel KL, Millard PR, Wojnarowska F. The treatment of vulval lichen sclerosus with a very potent topical steroid (clobetasol propionate 0.05%) cream. Br J Dermatol 1991;124:461–4. 2039723 10.1111/j.1365-2133.1991.tb00626.x

[8] Lee A, Bradford J, Fischer G. Long-term Management of Adult Vulvar Lichen Sclerosus: A Prospective Cohort Study of 507 Women. JAMA Dermatol 2015;151:1061–7. 26070005 10.1001/jamadermatol.2015.0643

[9] Kirtschig G, Becker K, Günthert A, Evidence-based (S3) Guideline on (anogenital) Lichen sclerosus. J Eur Acad Dermatol Venereol 2015;29:e1–43. 10.1111/jdv.13136 26202852

[10] Dhurat R, Sukesh M. Principles and Methods of Preparation of Platelet-Rich Plasma: A Review and Author’s Perspective. J Cutan Aesthet Surg 2014;7:189–97. 25722595 10.4103/0974-2077.150734 PMC4338460

[11] Chicharro-Alcántara D, Rubio-Zaragoza M, Damiá-Giménez E, Platelet Rich Plasma: New Insights for Cutaneous Wound Healing Management. J Funct Biomater 2018;9:10. 10.3390/jfb9010010 PMC5872096 29346333

[12] Alves R, Grimalt R. A Review of Platelet-Rich Plasma: History, Biology, Mechanism of Action, and Classification. Skin Appendage Disord 2018;4:18–24. 29457008 10.1159/000477353 PMC5806188

[13] Everts P, Onishi K, Jayaram P, Lana JF, Mautner K. Platelet-Rich Plasma: New Performance Understandings and Therapeutic Considerations in 2020. Int J Mol Sci 2020;21:7794. 10.3390/ijms21207794 PMC7589810 33096812

[14] Behnia-Willison F, Pour NR, Mohamadi B, Use of Platelet-rich Plasma for Vulvovaginal Autoimmune Conditions Like Lichen Sclerosus. Plast Reconstr Surg Glob Open 2016;4:e1124. 27975027 10.1097/GOX.0000000000001124 PMC5142493

[15] Casabona F, Gambelli I, Casabona F, Santi P, Santori G, Baldelli I. Autologous platelet-rich plasma (PRP) in chronic penile lichen sclerosus: the impact on tissue repair and patient quality of life. Int Urol Nephrol 2017;49:573–80. 28161837 10.1007/s11255-017-1523-0

[16] Goldstein AT, King M, Runels C, Gloth M, Pfau R. Intradermal injection of autologous platelet-rich plasma for the treatment of vulvar lichen sclerosus. J Am Acad Dermatol 2017;76:158–60. 27986140 10.1016/j.jaad.2016.07.037

[17] Franic D, Iternička Z, Franić-Ivanišević M. Platelet-rich plasma (PRP) for the treatment of vulvar lichen sclerosus in a premenopausal woman: A case report. Case Rep Womens Health 2018;18:e00062. 29785390 10.1016/j.crwh.2018.e00062 PMC5960026

[18] Goldstein AT, Mitchell L, Govind V, Heller D. A randomized double-blind placebo-controlled trial of autologous platelet-rich plasma intradermal injections for the treatment of vulvar lichen sclerosus. J Am Acad Dermatol 2019;80:1788–9. 30639885 10.1016/j.jaad.2018.12.060

[19] Posey LK (2019). Evaluating in office surgery followed by platelet rich plasma to treat lichen sclerosus. Abstracts, Journal of Lower Genital Tract Disease: October 2019 - Volume 23 - Issue 4S - p S37–S81 doi: 10.1097/LGT.0000000000000491

[20] Tedesco M, Pranteda G, Chichierchia G, The use of PRP (platelet-rich plasma) in patients affected by genital lichen sclerosus: clinical analysis and results. J Eur Acad Dermatol Venereol 2019;33:e58–9. 30051614 10.1111/jdv.15190

[21] Tedesco M, Garelli V, Bellei B, Platelet-rich plasma for genital lichen sclerosus: analysis and results of 94 patients. Are there gender-related differences in symptoms and therapeutic response to PRP? J Dermatolog Treat 2020;:1–5. 10.1080/09546634.2020.1854650 33226278

[22] Jain NK, Gulati M. Platelet-rich plasma: a healing virtuoso. Blood Res 2016;51:3–5. 27104183 10.5045/br.2016.51.1.3 PMC4828525

[23] Eshtiaghi P, Sadownik LA. Fact or Fiction? Adipose-Derived Stem Cells and Platelet-Rich Plasma for the Treatment of Vulvar Lichen Sclerosus. J Low Genit Tract Dis 2019;23:65–70. 30252710 10.1097/LGT.0000000000000440

[24] Kwok R, Shah TT, Minhas S. Recent advances in understanding and managing Lichen Sclerosus. F1000Res 2020;9:369. 10.12688/f1000research.21529.1 PMC7233179 32518626

[25] Kravvas G, Shim TN, Doiron PR, The diagnosis and management of male genital lichen sclerosus: a retrospective review of 301 patients. J Eur Acad Dermatol Venereol 2018;32:91–5. 28750140 10.1111/jdv.14488

[26] Rehman J, Traktuev D, Li J, Secretion of angiogenic and antiapoptotic factors by human adipose stromal cells. Circulation 2004;109:1292–8. 14993122 10.1161/01.CIR.0000121425.42966.F1

[27] Boero V, Brambilla M, Sipio E, Vulvar lichen sclerosus: A new regenerative approach through fat grafting. Gynecol Oncol 2015;139:471–5. 26499935 10.1016/j.ygyno.2015.10.014

